# Assessing the accuracy of CMRtools software for diagnosing liver iron overload in thalassemia patients: influencing factors and optimisation strategies

**DOI:** 10.3389/fmed.2024.1424294

**Published:** 2024-09-20

**Authors:** Chaotian Luo, Fei Peng, Fengming Xu, Cheng Tang, Yanyan Zhang, Chaojie Huang, Linlin Liang, Xiaojing Ning, Peng Peng

**Affiliations:** ^1^Department of Radiology, The First Affiliated Hospital of Guangxi Medical University, Nanning, China; ^2^NHC Key Laboratory of Thalassemia Medicine, Guangxi Medical University, Nanning, China

**Keywords:** liver, iron overload, magnetic resonance imaging, T2* relaxometry, CMRtools, thalassemia

## Abstract

**Background:**

CMRtools is a software package that can be used to measure T2* values to diagnose liver iron overload, however, its accuracy in terms is affected by multiple factors, including goodness-of-fit (R^2^ value), the number of echo time (TE) images, and the liver iron concentration (LIC). To investigate the effects of the R^2^ value, the number of TE images, and the LIC on the accuracy of CMRtools software for measuring T2* values to diagnose liver iron overload (LIO).

**Materials and methods:**

CMRtools software was used to measure liver T2* values among 108 thalassemia patients via the truncation method, and the R^2^ values, the number of TE images, and T2* values were recorded. These values were subsequently converted into liver iron concentration (LIC_T_) values. The LIC_F_ (derived from MRI-R2/FerriScan) was used as a reference, and the diagnostic accordance rate (DAR) was compared between R^2^ value subgroups, between TE image number subgroups, and between LIC subgroups.

**Results:**

The greater the R^2^ value was, the greater the standardized DAR (SDAR) was (*p* < 0.05). The SDAR are not identical between each TE image number subgroup (*p* > 0.05). However, the relationship between TE image number subgroups and SDAR was analysed using Spearman’s correlation, and it was found to be positively correlated (*r_s_* = 0.729, *p* = 0.017). The SDAR are not identical between each LIC subgroup (*p* > 0.05), furthermore, the relationship between LIC subgroup and SDAR was found irrelevant (*p* = 0.747).

**Conclusion:**

The accuracy of CMRtools software for diagnosing LIO in patients with thalassemia can be improved by artificially controlling the number of TE images to be fitted and selecting higher R^2^ values.

## 1 Introduction

Thalassemia is a group of inherited haemolytic anaemia disorders that are caused by deficiencies or insufficiencies in the synthesis of the globin chain (1). Iron overload of the organs—especially the liver—can occur due to increased intestinal absorption of iron in thalassemia patients due to ineffective haematopoiesis or due to the requirement for regular or irregular transfusion therapy (2, 3). Magnetic resonance imaging (MRI) is a sensitive and stable method for evaluating the liver iron concentration (LIC). Additionally, the use of relaxometry based on T2/R2 and T2*/R2* has been widely validated (4–6). A commercial method based on T2/R2 relaxometry (FerriScan. Resonance Health) was approved by the U.S. Food and Drug Administration (FDA) (7–9). However, T2*/R2* relaxometry is a more widely used method in clinical practice (10, 11), the reason for this phenomenon is that the T2*/R2* relaxometry has a shorter scanning time compared to T2/R2 relaxometry, the program is simple and inexpensive (12, 13).

CMRtools software (CMRtools/Thalassemia Tools, Cardiovascular Imaging Solutions, London, United Kingdom) is one of the most commonly used tools for measuring T2* in the clinic (14). This software used nonlinear least squares fitting to measure T2*. The measurement interface displays the goodness of fit (usually assessed using the R^2^ value) and the fit curve. The user can change the R^2^ value by the “truncation method” (15)—i.e., removing images with long TE (from large to small TE values), selecting the appropriate R^2^ value to determine the final T2* value, and converting the T2* value to the LIC_T_ according to the formula (16, 17). Nevertheless, when CMRtools are used to measure T2* values, factors such as the echo time, MR field strength, degree of LIC, image quality, and selection of the region of interest (ROI) may affect the accuracy of the measurements (18). The calculation of T2* value is based on the fitting of multiple TE signals, and the number of TEs affects the fitting (19). The decay rate of the T2* signal is positively correlated with the MR field strength and LIC (20), excessive signal decay could cause a large error in the calculated T2* value or even the inability to calculate (21). The poor quality of image may cause the ROI to contain non-hepatic tissue, thus affecting the judgment of the iron content of the liver parenchyma, etc. Thereby affecting the choice of clinical iron chelation treatment protocols, increasing the risk of organ function injury due to iron toxicity in patients with thalassemia, and potentially threatening the lives of these patients.

To date, few studies have comprehensively investigated the effect of CMRtools on the diagnosis of liver iron overload (LIO) in thalassemia patients. Thus, we used the CMRtools truncation method to obtain different R^2^ values, and we recorded the corresponding T2* values to further calculate the LIC_T_, which was compared with the LIC_F_ (derived from MRI-R2/FerriScan) to explore the effects of R^2^ values, the number of TE images, and the LIC, so that assess the accuracy of CMRtools to diagnose LIO in thalassemia patients.

## 2 Materials and methods

### 2.1 Study participants

A retrospective cohort of 305 patients who underwent MRI to measure iron in the liver at our medical center between 1 January 2018 and 31 December 2022 was included. The inclusion criteria were as follows: (i) had a genetic diagnosis of thalassemia; (ii) had complete MRI-T2* sequence images; (iii) had MRI-R2/FerriScan LIC results; (iv) had a history of regular or irregular blood transfusion; and (v) had simultaneous MRI-T2* and MRI-R2/FerriScan scans performed using the same instrument. The exclusion criteria were as follows: (i) patients with chronic diseases of the liver, such as cirrhosis, hepatocellular carcinoma or liver-occupying lesions; and (ii) patients with significant artefacts on the image that affected the measurement. All patients (or their parents/guardians) provided written informed consent to participate in this study. The study was conducted in accordance with the principles of the Declaration of Helsinki and approved by the Ethics Committee (NO.2024-E196-01). Ultimately, 108 patients were included in this study (Table 1).

**Table 1:** Study participant characteristics of 108 patients.

Characteristic	Value
Age, (y)*	17.61 ± 7.801
**Sex, *n* (%)**	
Female	48 (44.44)
Male	60 (55.56)
LIC, mg/g dry weight	0.6–36.2
**LIC degree, *n* (%)**	
Normal	12 (11.11)
Slight	15 (13.89)
Mild	36 (33.33)
Moderate	33 (30.56)
Severe	12 (11.11)

LIC represents liver iron concentration; *Date are the means ± standard deviation.

### 2.2 MRI acquisition

Liver MRI was performed on a 1.5 T scanner (MAGNETOM Avanto Fit, Siemens Healthcare, Erlangen, Germany).

MRI-R2/FerriScan acquisition consisted of a free breathing 2D multi-slice spin-echo pulse sequence. The relevant pulse sequence parameters were as follows: flip angle = 90°; echo time (TE) = 6, 9, 12, 15, 18 ms; repetition time (TR) = 1,000 ms; FOV read = 400 mm × 400 mm; matrix = 256 mm × 256 mm; and 11 slices with a thickness of 5 mm. Each TE acquisition time was approximately 1 min 40 s or less, and if one of the TE image artefacts was too large, five TE were rescanned. A reference was placed within the scanning field of view, usually a bag of normal saline.

T2* data were acquired using a breath-hold multi-echo GRE scanning sequence at the same liver level as FerriScan acquisition with free breathing. The relevant pulse sequence parameters were as follows: flip angle = 20°; echo time (TE) = 1.29, 2.35, 3.43, 4.60, 5.68, 6.85, 7.93, 9.10, 10.18, 11.35, 12.43, 13.6 ms; repetition time (TR) = 200.00 ms; FOV read = 400 mm × 400 mm; matrix = 256 mm × 256 mm; slice thickness = 10 mm; and scan time about 15 s.

### 2.3 Data processing

**Figure 1:**
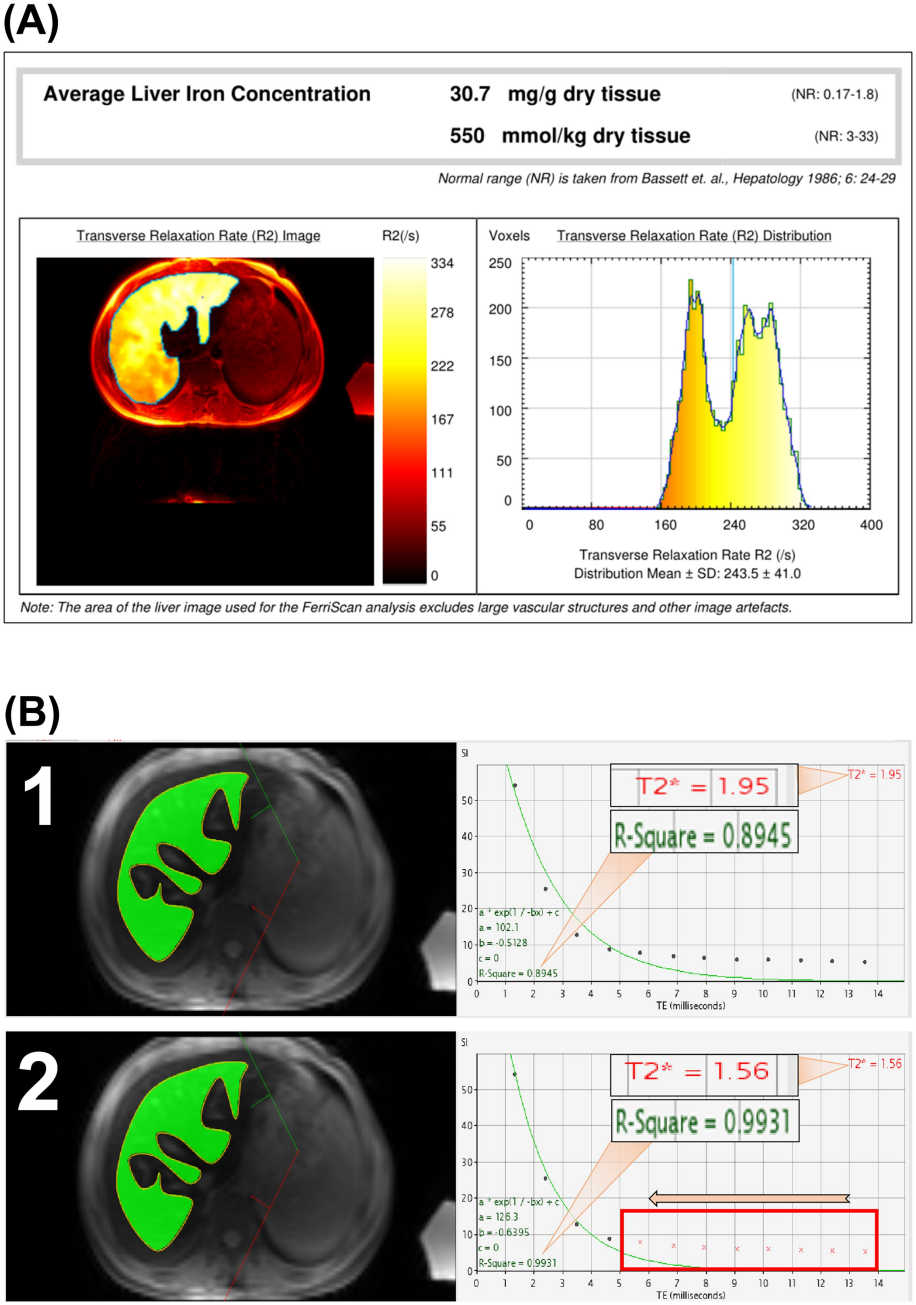
MRI-R2/FerriScan liver iron concentration report and schematic diagram of CMRtools measurements of liver iron concentration (LIC). Schematic diagram of LIC in an 18-year-old male patient with *β*-thalassemia major; the LIC_F_ was 30.7 mg/g dry weight **(A)**. The number of TE images entering the measurement was 12, with a T2* of 1.95 ms and an R^2^ value of 0.8945 **(B1)**, and the number of TE images with a large TE value of 6 was removed, resulting in the number of TE images entering the measurement being 4 (the red crosses in the box are the number of TE images removed), T2* is 1.56 ms, R^2^ value is 0.9931 **(B2)**, and both the T2* values and R^2^ value changed.


The liver MRI-R2 images were subsequently sent to the Resonance Health Data Processing Centre for processing, after which a LIC_F_ report was obtained (Figure 1A). The T2* data were imported in DICOM format into a computer with CMRtools software installed, and a radiologist with more than 5 years of experience in using software features used the CMRtools/thalassemia tools function and referenced to the MRI-R2/FerriScan ROI to outline the T2* ROI while avoiding visible bile ducts and blood vessels. The number of TE images, the T2* value and the R^2^ value were measured and recorded using the truncation method (Figure 1B). Finally, the LIC_T_ was calculated by substituting the T2* value into Garbowski’s (16) formula. Garbowski’s formula is as follows:

$$LIC=31.94•T2{*}^{-1.014}$$

where LIC is the liver iron concentration in mg/g dry weight, and the T2* value in ms. Groupings were made according to LIC (regardless of the method used to obtain LIC) and with reference to Reeder’s review (5) (Table 2). The R^2^ values from 0.9500 to 1.0000 were divided into 5 subgroups with a spacing of 0.01, and the number of TE images (ranging from 3 to 12) was divided into 10 subgroups.

**Table 2:** The degree of liver iron concentration.

Group	Liver iron concentration
Normal	< 1.8
Slight	1.8–3.2
Mild	3.2–7.0
Moderate	7.0–15.0
Severe	> 15.0

Liver iron concentration in mg/g dry weight.

### 2.4 Statistics

Statistical analysis was performed using the SPSS 27.0 (IBM, Armonk, New York). The R^2^, T2*, and LIC_F_ values did not follow a normal distribution. The paired samples Wilcoxon signed rank test was used to explore the differences in these variables. A *p* value >0.05 indicated no statistically significant difference. The intraclass correlation coefficient (ICC) was used to evaluate the consistency level. An ICC > 0.75 and a *p* < 0.05 were considered to indicate a high degree of consistency. Spearman’s correlation analysis was used to examine correlations. Correlation coefficients were considered strong when |*r_s_*| > 0.75 and *p* < 0.05. LIC_T_ and LIC_F_ values were grouped and described using the number of patients, the percentage, and the constituent ratio.

The accuracy of the CMRtools software was assessed using the diagnostic accordance rate (DAR), which was calculated by comparing the LIC_F_ grade to the LIC_T_ grade, counting the number of correct patients with consistent grades, and then dividing the number of correct patients by the total number of analyses. This study focused on three variables; therefore, when analyzing the effect of one variable on the DAR, the other two variables were artificially fixed at the same level. When comparing the DARs of a subgroup, each subgroup contains two other variable subgroups with different constituent ratios. Therefore, the standardized diagnostic accordance rate (SDAR) was used for comparison. The SDAR was calculated via the direct method: (i) the patients in the same layer in each comparison subgroup were combined to determine the number of patients in the standard group; (ii) the original DAR for the same layer in each comparison subgroup was determined; and (iii) the values were inserted into Formula. The formula is as follows:


$$p^{\prime }=\sum \left(\frac{Ni}{N}\right)\mathrm{pi}$$

where *p*′ is the SDAR, N is the total number of cases in the standard group, *Ni* is the number of cases in a layer in the standard group, and *pi* is the original DAR in each layer in the comparison subgroup. Differences between the SDAR were assessed using the Wilcoxon test; for multiple independent SDAR, differences were assessed using the Kruskal Wallis H test, and *p* values were calibrated using Bonferroni’s method when making multiple two-by-two comparisons.

## 3 Results

### 3.1 Analysis of measurement data

**Figure 2:**
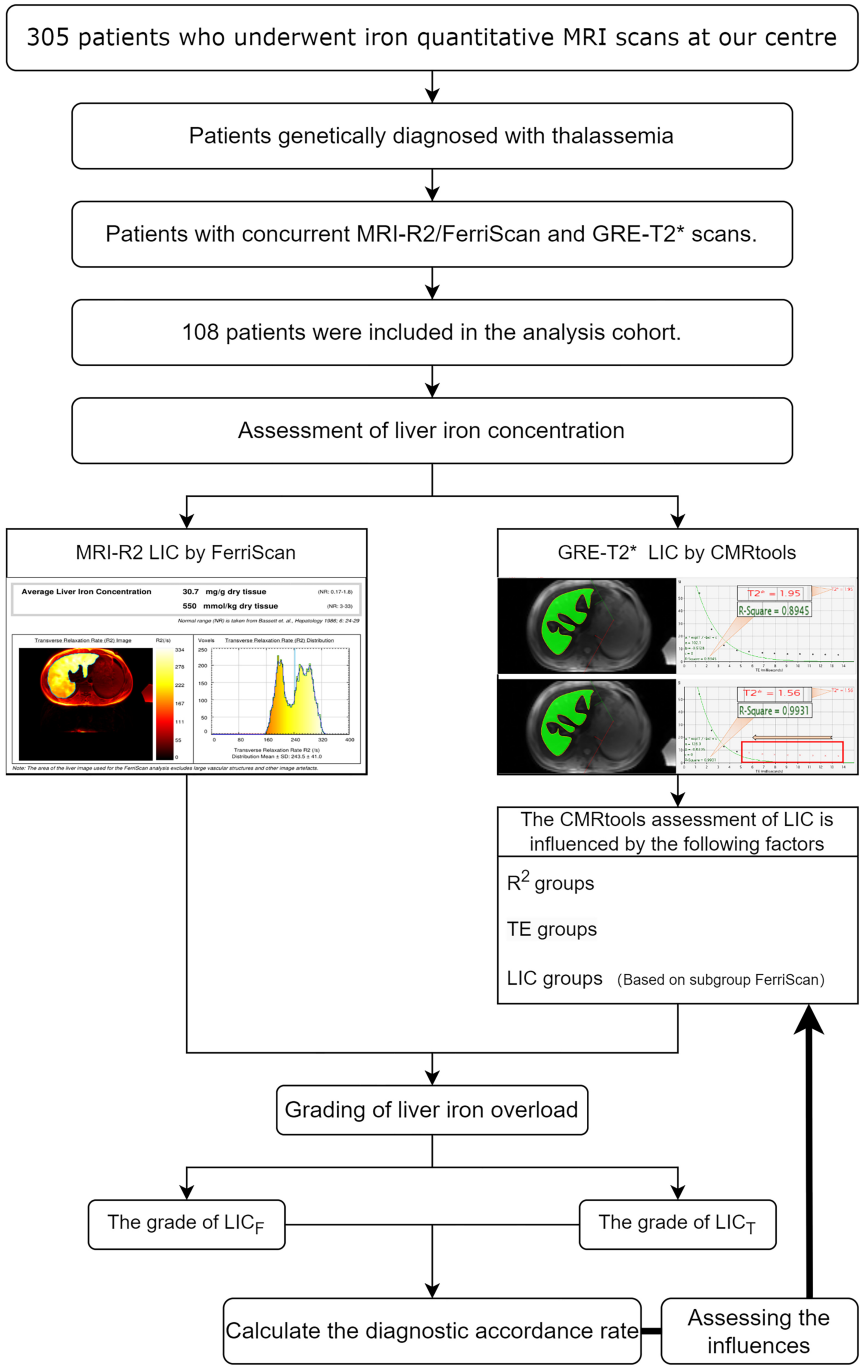
Schematic diagram of this study. MRI-R2 LIC_F_ data and T2* values were calculated using CMRtools software, the T2* value was converted to LIC_T_ for 108 thalassemia patients, and the accordance rate was calculated after grouping the LIC. The standardized diagnostic accordance rates among the R^2^ subgroups, among the TE duration subgroups, and among the LIC subgroups were analysed by using the controlled variable method.

A total of 108 patients were obtained using a truncation method of 1,080 R^2^ values and 1,080 T2* values, and 1,069 patients were obtained after excluding those whose R^2^ values were less than 0.95 (see Figure 2). Thirty-five percent of the data were randomly selected for reliability analyses between measurements by different measurers and between measurements by the same measurer at different times (2-week intervals) using the blinded method, resulting in *Z* = −0.047 (*p* = 0.963) and ICC = 0.991 (*p* < 0.001), indicating a high degree of consistency. The results of the analyses across different measurers were *Z* = −0.130 (*p* = 0.897) and ICC = 0.992 (*p* < 0.001), indicating a high degree of consistency.

### 3.2 SDAR analysis of the R^2^ value groups

**Figure 3:**
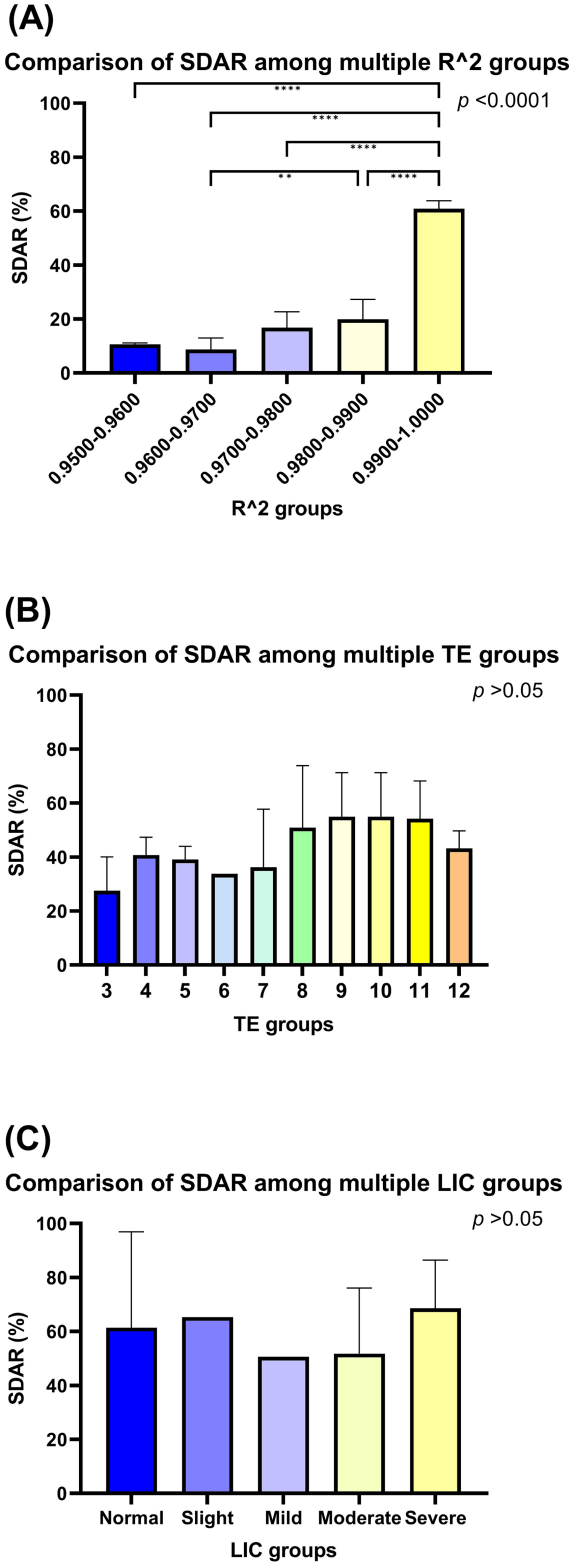
Comparison of standardized diagnostic accordance rate among groups. The *p* values in the upper right corner of the figure represent the original hypothesis, where H_0_ is the *p* value for all groups identical SDARs. Two-by-two comparisons between groups are linked by a horizontal line and labeled with “*,” with one or more “*” indicating that the corrected *p* value for two-by-two comparisons between groups using the Bonferroni’s method is less than 0.05. R^2^ is not identical between subgroups, and it is highest at 0.9900–1.0000, which is significantly different from all other groups **(A)**. Among TE subgroups, it cannot yet be denied that all groups are not identical **(B)**. Similarly, among LIC subgroups, it cannot yet be denied that all groups are not identical **(C)**.

A comparison of the SDAR of each R^2^ value subgroup showed that the highest SDAR was observed in the 0.9900–1.0000 subgroup, and the lowest SDAR was observed in the 0.9500–0.9600 subgroup. The R^2^ groups are not identical (*p* < 0.0001), and the 0.9900–1.0000 subgroup is not identical to the other groups (all *p* < 0.05). The SDAR for each TE image number subgroup increased with increasing R^2^ value, and the mean SDAR for each TE image number subgroup was positively correlated with the R^2^ value (*r_s_* = 0.900, *p* = 0.037) (Table 3, Figure 3A).

**Table 3:** The relationship between variable and SDAR.

Variable pairs	*r*	*p*-value
SDAR & R^2^ groups	0.900	0.037
SDAR & TE groups	0.729	0.017
SDAR & LIC groups	0.200	0.747

*r represents Spearman’s correlation coefficient and SDAR represent standardized diagnostic accordance rate.*

### 3.3 SDAR analysis of TE image number groups

The SDAR are not identical between each TE image number subgroup (*p* > 0.05). However, the relationship between TE image number subgroups and SDAR was analysed using Spearman’s correlation, and it was found to be positively correlated (*r_s_* = 0.729, *p* = 0.017) (Table 3, Figure 3B).

### 3.4 SDAR analysis of LIC groups

The SDAR are not identical between each LIC subgroup (*p* > 0.05), furthermore, the relationship between LIC subgroup and SDAR was analysed using Spearman’s correlation, and it was found irrelevant (*p* = 0.747) (Table 3, Figure 3C).

## 4 Discussion

For a better comparison, this study used SDAR for comparison, and SDAR effectively avoids the embarrassment of having different internal composition ratio between comparison groups and achieves the removal of non-analytical factors. For example, when analyzing the SDAR comparison between the R^2^ groups, the TE subgroup grades and LIC subgroup grades within each of the R^2^ groups were different, and the first step of the SDAR was to combine the TE subgroups and record them as the standardized group number, and recalculate the DAR by multiplying its original DAR by the standardized group number, which eliminated the influence of the composition ratio of the TE groups as much as possible, and then calculate the SDAR of each LIC subgroup after the SDAR of each group mean, further minimizing the effect of LIC, and finally in making a more reasonable comparison between the R^2^ groups, the utilization of SDAR was an ingenious use.

This study focused on the analysis of three factors affecting the diagnostic LIO of CMRtools. Although the only controllable variables are the TEs, both R^2^ and T2* vary depending on the chosen TEs. The R^2^ values may differ for the same TEs between patients; it is possible for R^2^ to be higher for a smaller number of TEs, or to fit well for a larger number of TEs. Consequently, with varying R^2^ values, T2* also varies. To standardize the measurement of T2*, we conducted the following analysis.

The first factor was the goodness-of-fit R^2^ value. CMRtools was used to measure T2* values obtained by nonlinear least squares fitting of multiple consecutive TE image signals, and R^2^ values are a statistical indicator used to reflect the degree of reliability of the regression model in describing changes in the dependent variable. Higher R^2^ values indicate that the independent variable can explain a greater proportion of variance in the dependent variable (22). Due to the different constituent ratios of different LIC subgroups and TE image number subgroups within each R^2^ value group (a similar situation exists in analyzing the LIC subgroups and the TE image number subgroups). In this study, the R^2^ value was positively correlated with the SDAR, the results suggest that the higher the R^2^ value is, the greater the diagnostic accuracy is.

The second factor is the number of TE images. For the different confounding factors affecting the accuracy of T2*, especially the noise substrate, background B0 field variations, and the presence of fat, the wide applied noise substrate correction techniques include the truncation method (23) and the bias fitting method (24). A comparison of these two methods was carried out by He et al. and they concluded that the mono-exponential-truncated method is more stable than the mono-exponential-biased method (15). Herein, we used the truncation method, which involves removing large images with low-signal-intensity TE values that may be hidden in the noise. After standardizing the DAR, each R^2^ groups’ SDAR not identical; however, the relationship between TE image number subgroups and SDAR was positively correlated, it suggests that the larger the number of TE image, the more stable the fit, making the diagnostic results more stable and accurate.

The third factor is the impact of the degree of LIC on diagnostic results. Severe LIO is a considerable challenge for MRI-T2* based diagnosis of LIO, mainly because the high LIC in severe LIO leads to rapid decay of the MR signal, and routine echo times may detect little or no signal (25). Nonetheless, in this study, we believe that LIC may not significantly affect the accuracy of diagnosis. This is because there is no statistical evidence to suggest that LIC influences the rate of correct diagnosis. Thus, it implies that the CMRtools remains applicable in clinical practice for diagnosing the degree of LIO, even in cases of severe LIO resulting in rapid signal attenuation. Summarizing the above three analyses, the accuracy of CMRtools can be improved by the truncation method—artificially control the number of TEs entering the fit, and thus increase the value of R^2^ as much as possible. However, a principle should be followed, in the same R^2^ value subgroup, maintain maximum number of TEs. In this study, the truncation method was able to improve the DAR, but the selection of ROIs still needs to be rigorously screened because the uneven distribution of iron deposition in the liver is objective (26).

The MRI-R2/FerriScan results indicated a strong correlation between the 1.5 T R2 data and the LIC when the LIC ranged from 0.3 to 42.7 mg/g (r = 0.98) (9). However, the ability of MRI-R2/FerriScan to measure iron levels may be affected by factors other than iron concentration, such as the micro-distribution of iron and the presence of fat; these effects have yet to be fully elucidated (5, 27). Since chemical shift artefacts caused by fat have been shown to affect R2* values (28), we adopted the strategy of fat suppression to minimize this effect. MRI-R2/FerriScan, which uses a spin-echo sequence, is not significantly affected by chemical shift artefacts (29). Doyle et al. (30) noted that the FDA-approved LIC_F_ was greater than the actual LIC at LIC > 16.5 mg/g dry weight. Moreover, the analysis of the data at LIC_F_ > 16.5 mg/g showed that 36.36% (4/11) of the data were classified as moderate LIO for LIC_T_ and severe LIO for LIC_F_, respectively. At LIC_F_ > 16.5 mg/g, it is possible to overestimate the actual LIC, although we do not know what the rationale is at this time.

Healy et al. suggested that within a certain range of T2*/R2*, relaxometry can be a substitute for T2/R2 relaxometry for the quantification of LIC (31). However, when heavy iron deposition occurs, the T2* signal decays faster, and small changes in T2* values can lead to significant changes in estimated iron values, which is a dilemma based on the use of T2*/R2* to diagnose ultra-severe hepatic iron deposition (32). Recently, several scholars have used MR-based ultrashort echo (UTE-T2*, UTE-QSM) sequences to overcome the acquisition drawbacks of T2* in diagnosing severe LIO by drastically shortening the time of the first TE, thus capturing the rapidly decaying signals of severe LIO; these studies have been well validated in animal models and in patients with iron overload (21, 33–37).

This study had multiple limitations. The sample size was too small. In addition, a single-center design was used, therefore, additional software for measuring T2* values, such as CVI42, the Func Tool, and Quanta Hematology, were not used for comparison. Diagnostic efficacy was mainly evaluated based on the SDAR, which can reduce the effect of different proportions of each layer within each comparison group to a certain extent and facilitate comparison but does not represent the actual diagnostic accuracy. The method for selecting the optimal ROI was not explored, mainly because of the specificity of iron deposition in the liver tissues of individual patients and the difficulty in choosing ROI locations in a uniform manner. Moreover, most of the MRI-R2/FerriScan ROIs did not include the whole liver.

## 5 Conclusion

CMRtools is a well-classified software package for LIC. The degree of LIO does not affect the correctness of the diagnostic accordance rate. Improving the accuracy of CMRtools in diagnosing LIO can be achieved by increasing the R^2^ value using the truncation method. At the same level of R^2^, the higher the number of TE image number, the higher the diagnostic accordance rate.

## Data Availability

The original contributions presented in the study are included in the article/Supplementary material, further inquiries can be directed to the corresponding author.
